# Italian Medical Congress

**Published:** 1888-12

**Authors:** A. Lagorio

**Affiliations:** Chiavari, Italy


### FOREIGN CORRESPONDENCE.

*To the Editor of the Medical Journal and Examiner:*

The annual meeting of the Italian Medical Congress was held in Rome on October 20, 22, 23, 24, and 25. Professor G. Baccelli, of Rome, acted as president, and Professor E. Maragliano, of Genoa, as secretary. The reunion was held in the University building, and was largely attended. President Baccelli delivered an address, dwelling especially on the visit of the Emperor of Germany, and on the duties of physicians in times of peace and war.

On October 22, a most thorough discussion was held on pneumonia. Professor Bozzolo, of Turin, spoke at length on its etiology. He mentioned the recent published stndies on the diplococcus of Fränckel, and on the streptococcus of Friedlander, and showed that the former is capable of giving origin to very different gradations of the disease. He admitted that other infective agents may determine pneumonic processes. He also demonstrated that the pulmonary cells offer a different resistance to the invasion of the diplococcus, according to the *circumambient* conditions.

Professor Magliano spoke on the treatment of pneumonia, regarding its etiology and pathogenesis. He admitted that pneumonia is an infective disease, and demonstrated that we must admit that the hypocinesis and the acinesis of the heart is due to the presence in the circulation of a toxic principle developed from the pathogenic micro-organisms. He did not approve the nihilism and the parenchymatous injections, and discussed the advisability of bleeding. To lower the fever, he advised the cold bath, and spoke against the objections raised against the refrigerant treatment. To avoid cardiac enfeeblement he avoided the use of ipecac, but prescribed alcohol, digitalis, and strophanthus.

Dr. Lucatello had observed, in examining the blood of pneumonics that the serum is absolutely sterile, and he attributed this to the development of a toxic substance from the patheogenic micro-organisms.

This important subject provoked interesting discussion from Professors Cardarelli, DeReuzi, Amoroso, De Giovanni, and others. Professor Cantani, of Naples, did not approve bleeding for the purpose of eliminating morbid poisons, and only rarely to empty a laboring heart. A physician must not treat the *pneumonia*, but the *patient* in general.

Professor A. Bianchi reported many of his researches, by which he had concluded that the heart in pneumonia dilates, and he therefore favored bleeding.

Professor Roucisvalle, of Catania, described several cases of infective pleurisy studied in his clinic.

In October 23, Professor Cardarelli, of Naples, spoke on the treatment of the functional disturbances and neuroses of the heart.

Professor De Giovanni, of Padua, mentioned the discussion held at the last congress at Wiesbaden on the cardiac gymnastics advocated by Hirtel. He said that the treatment of some cardiac disorders ought to be dietetic and gymnastic.

Professor Rummo explained the pharmaceutical therapy of the organic diseases of the heart, and demonstrated that the mechanism of treatment consists in proportionating the activity of the cardiac motor to the potency of the circulatory resistance with the least possible waste of strength.

In the afternoon Professor Murri, of Bologna, spoke at length on the genesis and the nature of fever, declaring himself against the majority of the modern scientists who believe it the effect of a mechanism always identical in different cases—that is, "the excitement of nervous centres as producers of the fever itself." He believes, instead, and demonstrates it on animals, that the nervous system of the patient is not the cause of the fever, but that the infecting material which enters the blood is. He therefore believes it impossible to speak of an unique treatment of fever, as there are as many fevers as infective diseases.

Professor Maragliano, of Genoa, did not agree with Murri about the mechanism of fever. He explained the good results which he had obtained with the moderate use of antifebrile remedies, also the advantages on the general condition of the patient, on his strength, etc.

Professor Cantani dissented from Maragliano. He combated the fever only in rare cases, and his ideal treatment would be to combat the cause, which we can not do at present, except in a few of the infective fevers.

Professor Baccelli, in resuming the discussion, said that he treated the fever, yet recognized that at times it ought to be let alone as beneficial to the organism. He favored cold ablutions, and recommended especially the application of ice in some regions of the body, as on the sides, and back of the neck.

On the 24th, Professors Cantani, Cardarelli, De Reuzi, Maragliano, Petri, and Rummo discussed the subject of diabetes and its treatment.

Dr. Peusuti made a full report of a case of an echinococcus cyst which he had in his clinic.

Professor Rovighi read a paper on amyotrophic sclerosis. He described his microscopical studies and reached important conclusions.

Dr. Lucatello discussed some muscular dystrophies, and showed several histological specimens.

Professor Fenoli described a case of poli-myositis, and presented some microscopical preparations.

Professor Cardarelli described a disease occurring in children, characterized by fever, enlargement of the spleen, and great emaciation. In one case the spleen was extirpated by Professor D'Antona with benefit to the patient.

Professor Ferretti reported the good effect obtained by introducing long needles into the spleen, with the view of diminishing its volume in malarial patients.

Professor Fazio, of Naples, had had analogous results with the injections of quinine in the spleen.

Several other subjects were discussed. Professor Amoroso spoke on the pulse, Dr. Testi on the treatment of typhoid fever, etc., etc.

On October 25, Dr. Guardi reported some good results obtained with the hypodermic injections of carbolic acid in some forms of neuralgia, as sciatica, superorbital and intercostal neuralgia, etc.

Professor Patella spoke of an epidemic of jaundice observed by him. He also showed some histological specimens of a case of chorea, in which little spots of softening would be seen, due to embolism of the capillaries of the encephalon.

A case of an intestinal invagination was next described by Dr. Ibba.

Dr. Serrafini spoke on the etiological unity of some forms of cerebro-spinal meningitis, pleurisy, pneumonia, and peritonitis, in which he found the etiological relation of these forms with the diplococcus of Fränckel.

At the afternoon session Professor Vizioli, of Naples, presented to the Congress an interesting hypnotic subject. The subject was a young Neapolitan lady who had received much benefit by hypnotism for the relief of hystero-epileptic convulsions, nervous asthma, and attacks of sleep which would last at times over two days. Professor Vizioli stated that he presented a case of great hypnotism, very rare, and such as has been described by Charcot, with the three classic stages, the lethargic, the cataleptic, and the somnambulic.

The young lady lay on a sofa, and by converging on her right eye the rays from a small mirror she was made to fall in the first stage of lethargy in one minute and nineteen seconds. While in this stage the professor provoked the following phenomena of neuro-muscular excitability: By touching the facial nerve the face contracted as if to laugh; by pressing on the cubital nerve the hand closed as if to grasp something, and opened again. Consciousness was totally lost.

From this stage the subject passed rapidly to the cataleptic state. By pressing on the occiput the subject fell into the somnambulic state. She was then suggested to have, after five minutes' time, a paralysis of motion, and of sensation of the right arm, also an anæsthesia of the pharynx.

The subject was wakened by blowing of breath in the eyes, and after five minutes, the paralysis of the arm was complete as was suggested. She was punctured with pins, also a flame of a lamp was put under the palm of her hand, yet she felt nothing. The pharynx was also proven to be anæsthetic.

Professor Vizioli desired to demonstrate by this experiment the existence of an hypnotism induced by somatic characters, as an organic form of neurosis, distinct from the neurosis due to psychical causes, —that is, by means of suggestion. He wanted to demonstrate that in determinated subjects the three stages naturally follow each other, without imposition of an external will, and with particular characters of flexibility, of muscular and nervous contractility, which, indeed, characterize the spontaneous nature.

Several other cases were reported to the society and discussed. The work of the Congress being ended, President Baccelli, after a neat speech of thanks, adjourned the meeting to 1889, to meet again in Rome on October 15, 16, 17, and 18.

Professor Guido Baccelli was again elected president; Professors A. Cantani, and A. Murri, vice-presidents; E. Maragliano, secretary, and E. Rossoni, treasurer.

Last Sunday, October 28, two monuments were dedicated at Milan. One was dedicated to the memory of the late Dr. Gaetano Pini, the founder of the Milan Rachitic Institute, of the Italian Society of Hygiene, and of the Cremation Society. The other was dedicated to the late Dr. Agostino Bertani. He was a patriot, a follower of Garibaldi, and took part in all the principal battles for the independence of his country. He was the author of an important sanitary codex, which has been unanimously approved at the last session of the Italian parliament. Both men have been deeply mourned as a great loss to their country, to humanity, and to science.

A. LAGORIO, M. D.

CHIAVARI, ITALY, October 31, 1888.

